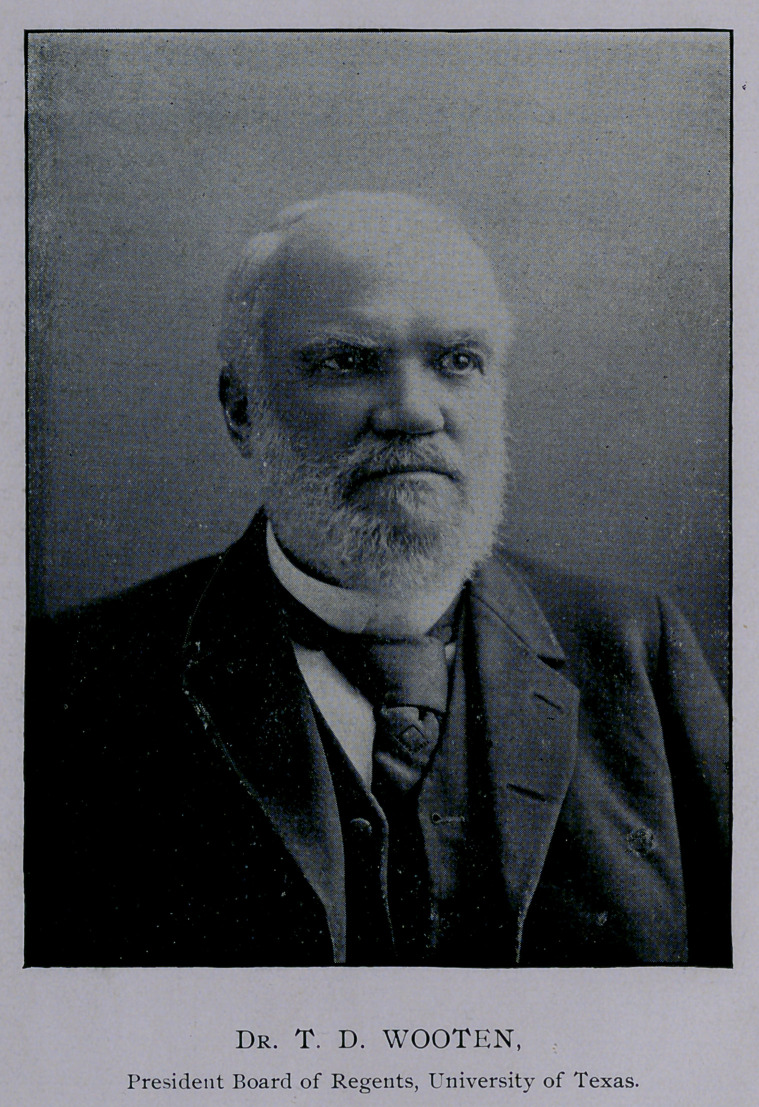# Biographical Sketches of the Faculty

**Published:** 1892-07

**Authors:** 


### Biographical Sketches of the Faculty.

### PROF. J. Y. F. PAINE, M. D., DEAN.

OBSTETRICS, ETC.

Dr. Paine, Professor of Obstetrics and Dean of the College, is a native of the Pelican State. He was born in West Feliciana Parish, La., August 16, 1840, and is of Scotch-English descent. He received an Academic education at Centenary College, Louisiana, and graduated in Medicine at the University of Louisiana, in 1861, during the service of the immortal Stone. On the breaking out of the war between the States, Dr. Paine entered as a private soldier in the 20th Louisiana Regiment of Volunteers; was appointed Assistant Surgeon of the 22nd Louisiana Regiment, December, 1861. After the fall of New Orleans, he served in the hospitals at Corinth and Holly Springs, Miss.; was examined May, 1862, at Columbus, Miss., by the Army Board of Medical Examiners (Yandell, Pim and Heustis), and, as stated, was commissioned Surgeon with rank and pay of Major of Cavalry; assigned as Surgeon 21st Alabama Regiment, which was sent to Fort Morgan, at the mouth of Mobile bay. By seniority of commission, he took rank as Chief Surgeon of the forces constituting the defense of Mobile bay. At the fall of these forts in '64, Dr. Paine was assigned as Chief Surgeon of General Hospital Nidelet, at Mobile, where he served till the surrender of Mobile, in '65. Hence, he was ordered to Gainsville, Alabama, and took rank as Surgeon in charge of the General Hospital at that Post, and remained there till the final surrender of all the Confederate forces, in June, 1865.

Upon the declaration of peace, Dr. Paine settled in Mobile, and engaged in general practice; removed to Texas in 1874; was elected to the Chair of Obstetrics and Diseases of Women and Children in the Texas Medical College and Hospital at Galveston, in 1875; after competitive examination, was made Dean of the Faculty in '79; was elected Chairman of the Section on Gynæcology in the Texas State Medical Association, in 1885, and Chairman of the Section on Practice, in 1886; was chosen Secretary to the Section on Gynæcology in the American Medical Association, 1885; elected President Galveston County Medical Society in the same year; was one of the Vice-Presidents of the Section on Public and International Hygiene of the Ninth International Medical Congress; elected to the Chair of Materia Medica, Therapeutics and Hygiene in the Medical Department of Tulane University—his *alma mater*, in 1885, which position he filled one term, to the entire satisfaction of the Faculty and Trustees, and with distinguished credit to himself and to Texas. Resigning this honorable position, for private reasons satisfactory to himself, he resumed practice in Galveston, where he has a large clientelle of the wealthier classes, and lives in elegance and comfort, in a beautiful home on Broadway—the Boulevard of Galveston—the fruits of his individual labors and industry. On resigning the chair in the University, at the close of the session, after repeated solicitations to reconsider his determination, he was made the recipient of a testimonial from the Faculty, in the shape of a set of resolutions, expressive of the high appreciation of his services (which were characterized as eminently satisfactory and valuable), entertained by his colleagues, individually and collectively; and of deep and sincere regret at the necessity which induced him to sever relations so pleasant to them. These resolutions bore testimony to Dr. Paine's professional attainments and ability, no less than to those agreeable social qualities for which he is distinguished; and altogether, expressed a sincere regard for him as a teacher, a physician and a man—whom to know, is to respect; couched in a language as courteous as complimentary.

Dr. Paine is an honorary member of the Southern Surgical and Gynæcological Association; an honorary member of the Louisiana State Pharmaceutical Association, and was President Texas State Medical Association in 1888-9. He filled the Chair of Obstetrics and Diseases of Women and Children in the Texas Medical College in 1888-9-90, and upon the organization of the Medical Department of the University of Texas in 1891, was chosen by the Regents for the corresponding position, and filled that chair the first session, 1891-2, having been also elected Dean, the position which he now fills. He has contributed but little to current medical literature, being kept busy by his large practice, the demands of which were such as to prevent his even being present in the hall when his election as President Texas State Medical Association was announced amidst cheers and applause. His best papers are to be found in the Transactions of the Texas State Medical Association—notably his address as

### STAFF OF SEALY HOSPITAL

AND

### FACULTY MEDICAL DEPARTMENT UNIVERSITY OF TEXAS

GALVESTON, TEXAS.

PROF. J. E. THOMPSON.

PROF. A. J. SMITH.

PROF. EDWARD RANDALL.

PROF. H. A. WEST.

PROF. SETH M. MORRIS.

PROF. WM. KEILLER.

PROF. A. G. CLOPTON.

Chairman of the Section on Practice—and in the New Orleans *Medical and Surgical Journal.*

Dr. Paine is characterized by a distinguished courtesy of manners, and has an easy and forcible manner of speaking which is impressive. These conjoined to a splendid physique mark him as a man well fitted to lead, and especially adapted to the position he fills.—D.

---

### PROF. H. A. WEST, M. D.

### PRACTICE OF MEDICINE.

Hamilton Atcheson West is of Scotch-Irish, Huguenot and English ancestry; born March 30, 1849, at Rupells Cave, Fayette county, Kentucky; was graduated in medicine from Medical Department University of Louisville, Ky., 1872, taking highest honor in a graduating class of 98. Elected by competitive examination one of the house-surgeons of the Louisville City Hospital, April, 1872. Came to Texas in 1873; elected in the autumn of that year Professor Materia Medica and Therapeutics, Texas Medical College. Appointed house surgeon of the Galveston City Hospital, in the spring of 1874; reappointed to the same position in 1877. At the reorganization of the Texas Medical College, 1888, he was elected Professor of Theory and Practice of Medicine. Elected Fellow of American Association of Gyneocologists and Obstetricians in 1889. Elected Secretary of the State Medical Association, April, 1891. Elected Professor of Theory and Practice of Medicine, and Clinical Medicine, June, 1891, in School of Medicine, University of Texas—the position he now holds.

---

### PROF. E. RANDALL, M. D.

### MATERIA MEDICA AND THERAPEUTICS.

Edward Randall was born in Walker county, Texas, October 7th, 1860, of a long line of medical ancestry.

He received his academic education in Virginia, and was graduated from Washington and Lee University of that State in 1889. He entered the Medical Department of the University of Pennsylvania in 1880, and received the diploma of Doctor of Medicine in 1883.

He was resident physician in the Philadelphia Hospital

(Blockley) for one year, and from there he entered the European schools, studying under Virchow and E. Martin in Berlin, Winckel in Munich. Carl Braun and Billroth in Vienna. He began the practice of medicine in Galveston in 1886. He was elected to fill the chair of Materia Medica and Therapeutics in the Texas Medical College and Hospital in 1888, and in 1891 was elected to the same chair in the Medical Department of the University of Texas.

---

### PROF. WILLIAM KEILLER, M. D.

### ANATOMY.

---

William Keiller, L. R. C. P. & S. Ed. L. F. P. & S. G. F. R. C. S. Ed., Professor of Anatomy, Medical Department University of Texas, was born in Midlothian, Scotland, on the 4th of July, 1861; educated in Perth Academy, and afterward in Edinburgh University; studied medicine in Edinburgh University and the Edinburgh Medical School. While a student he obtained the senior silver medal for Practical Anatomy and was Pattison prizeman for the best mounted dissection. He was successively Prosector, Junior and finally Second Senior Demonstrator of Anatomy to Dr. Macdonald Brown, from whom he received his anatomical training. In July, 1888, he obtained the conjoined diploma of the Royal College of Physicians and Surgeons of Edinburgh, and of the Faculty of Physicians and Surgeons of Glasgow, and in July, 1890, was elected Fellow of the Royal College of Surgeons of Edinburgh. He has been, successively, Demonstrator of Pathology under Dr. Alex. Bruce; House Surgeon at the Edinburgh Royal Infirmary, and Chloroformist to the Edinburgh Dental Hospital. He was assistant medical officer, and afterwards physician for diseases of women to the Edinburgh Provident Dispensary. In 1890 he was appointed Lecturer on Anatomy in the Edinburgh Medical School and elected Fellow of the Edinburgh Obstetrical Society.

---

### PROF. A. G. CLOPTON, M. D.

### PHYSIOLGY.

---

Dr. A. G. Clopton is a native of Georgia. He received a liberal education, both scientific and classical. Studied medicine and graduated M. D. from the medical department University of

Louisiana (now Tulane University), session of 1851-2. He located first at Camden, Arkansas, and entered upon the practice of medicine, and removed thence, in 1854, to Texas, settling in Cass county. Here, in connection with his practice, he engaged in farming. . . . In 1869, removed to Jefferson, Texas, and engaged in general practice of medicine and surgery, soon taking a leading position, which he held up to the time when he was chosen by a very discriminating Board of Regents to fill the chair of physiology in the medical department of the Texas State University, in 1891. Upon the breaking out of the war in 1861, Dr. Clopton raised a company of infantry, and at their head entered the Confederate service. From captain he was promoted to major of 1st Texas infantry. He went before the Board of Medical Examiners in 1863, and passing a rigid examination, was commissioned surgeon, serving in that capacity till the close of the war. He was married in 1854, to Miss Annie M. Henderson, during his residence in Cass county.

Dr. Clopton is an old member of the Texas State Medical Association, and was one of its first presidents, having filled that position in 1875. He was also President of the East Texas Medical Association in 1891, at the time of his election to the chair of physiology. He was one of the best known physicians in the State, and is famous as an extemporaneous speaker, possessing oratorical powers of a high order of excellence.

---

### PROF. SETH MABRY MORRIS, M. D.

CHEMISKTY.

Seth Mabry Morris, B. S., M. D., Professor of Chemistry Medical Department University of Texas, was born in Austin in 1867. His parents are Dr. W. A. Morris and Lucinda Mabry Morris. He received his preliminary education in the schools of Austin, and on completion of the University of Texas, matriculated amongst the very first pupils. He took the five year's course, devoting special attention to chemistry under Profs. Mallett and Everhart, and Physics under Dr. Macfarlane. In both these branches he won distinction, and during his last senior year he was chosen by Prof. Everhart as laboratory assistant. Graduating at the University in 1888, with the degree of B. S., he at once began the study of medicine in his father's office, and in the fall of that year entered the College of Physicians and Surgeons in

New York. Here he took the required three years course, giving special attention to chemistry under the instruction of Professor Chandler, and graduated M. D. from that school in 1891. In addition to the degree M. D., conferred upon him, he was awarded a "special examination diploma" and a cash prize, being one of the ten to whom special honors were awarded, in a graduating class of one hundred and fifty odd.

---

### PROF. J. E. THOMPSON, M. D.

### SURGERY.

James Edwin Thompson, age 28, was born in Northwich, England, and was educated at the Owens College, Manchester, England; obtained the scholarship and gold medal in anatomy at the London University and the Bradley and the Dunville surgical scolarships in connection with the Manchester School of Medicine; was admitted as a member of the Royal College of Surgeons in 1886, and a Fellow of the same college in 1888; obtained the degree of Bachelor of Medicine and the degree of Bachelor of Surgery of the London University, in both of which examinations he was placed in the honors list. He has held the posts of House Surgeon to the Royal Infirmary, Manchester; House Surgeon to the Dudley Hospital, England, and lastly, to the important post of Resident Surgeon to the Manchester Royal Infirmary, where he obtained his experience in teaching.

He studied on the continent, spending six months in Vienna and six months in Paris.

From some dozen or more applicants, Dr. Thompson was chosen by the Regent for the chair of Surgery in the Texas Medical College—he presenting the highest and most satisfactory credentials, and testimonials from the most eminent surgeons and anatomists in England, France, and Germany.

---

### ADJUNCT PROFESSORS.

In addition to the Faculty, Dr. R. C. Hodge, on Opthalmology; Dr. R. W. Knox, on Dermatology, and Dr. H. C. Cook, on Diseases of Children, were elected lecturers, and filled those positions during last session. There are other lecturers, but we have not been enabled to get their names; they appear in the catalogue, which can be had on application either to the President of the Board of Regents, or to the Dean.

### DR. T. D. WOOTEN,

### PRESIDENT BOARD REGENTS, UNIVERSITY OF TEXAS.

---

Thomas Dudley Wooten was born in Kentucky, March 6, 1829, of Virginia parents who settled in Kentucky in the early days. He was the youngest but one of several sons. At fifteen he was left by the death of his father master of a large farm and slaves. He received such education as the schools of the country afforded, aided by diligent reading at night and in the interval of labor. Studied medicine with Dr. George Rogers at Glasgow, Ky.; entered the Medical Department of the University of Louisville in the fall of 1851, when Gross, the elder Flint, Yandell, Sr., Drake, and other distinguished men were in the zenith of their fame, and graduated in the spring of 1853. Before graduating he was married to Miss Henrietta C. Goodall, daughter of Dr. Turner Goodall, of Kentucky. Located at the town of Tompkinsville, Ky., and entered upon an active practice. In 1856 removed to Springfield, Mo. Doing a general practice he had a natural fondness for surgery and soon acquired distinction in that branch.

On the breaking out of the war and the transfer of the Missouri troops (in which he had enlisted as a private) to the Confederate army, Dr. Wooten was chosen by the medical staff for Medical Director of the First Army Corps (composed of Missouri and Arkansas troops), and took rank as such on the staff of Major-General Sterling Price, commanding. Upon the transfer of this command to the east side of the Mississippi river, when Gen. Price was placed in command of the Department of Tennesssee, Mississippi, Louisiana and part of Alabama, Dr. Wooten was made Medical Director of the Department. Gen. Price being transferred to the West, in command of the District of Arkansas, Dr. Wooten retained position on his staff and served till end of the war. His rise and sustained success in the army were remarkable. Being only 32 years of age at the outbreak, he rose from private to Medical Director without prestige or influence, and in competition with some of the most eminent and influential men in St. Louis and the West.

On the cessation of hostilities Dr. Wooten, ruined in fortune, settled in the village of Paris, Texas, in 1865. Here he soon built up a fine practice and recuperated his fortune. Removed to Austin in 1876, and has continuously resided there to date. His reputation and success as a surgeon are part of the history of the medical profession of Texas. He is a prominent member of the Texas State Medical Association, the American Public Health Association,—and was a delegate to the Ninth International Medical Congress at Washington. Upon the organization of the Austin District Medical Association, in 1887, he was elected President. Upon the inauguration of the University of Texas, in 1881, Dr. Wooten was appointed by Gov. Roberts one of the original Regents, and reappointed by Gov. Ireland. In January, 1886, upon the death of Dr. Ashbel Smith, Dr. Wooten was unanimously elected President of the Board of Regents, which position he still holds. From the first he has been one of the most active and earnest friends of the University and has labored for its successful establishment with a zeal and fidelity that have faltered under none of the discouraging indifference and even hostility to the State's great seat of learning.

To Dr. Wooten the people of Texas owe a debt of gratitude; he has been the steadfast friend of education; and to him is also, in a large measure, due the successful inauguration of our high grade Medical Branch, which, in time, will be universally recognized as an honor to the great State of Texas. He and his able colleagues have carried out what the great founders of the commonwealth conceived and foreshadowed.

## Figures and Tables

**Figure f1:**
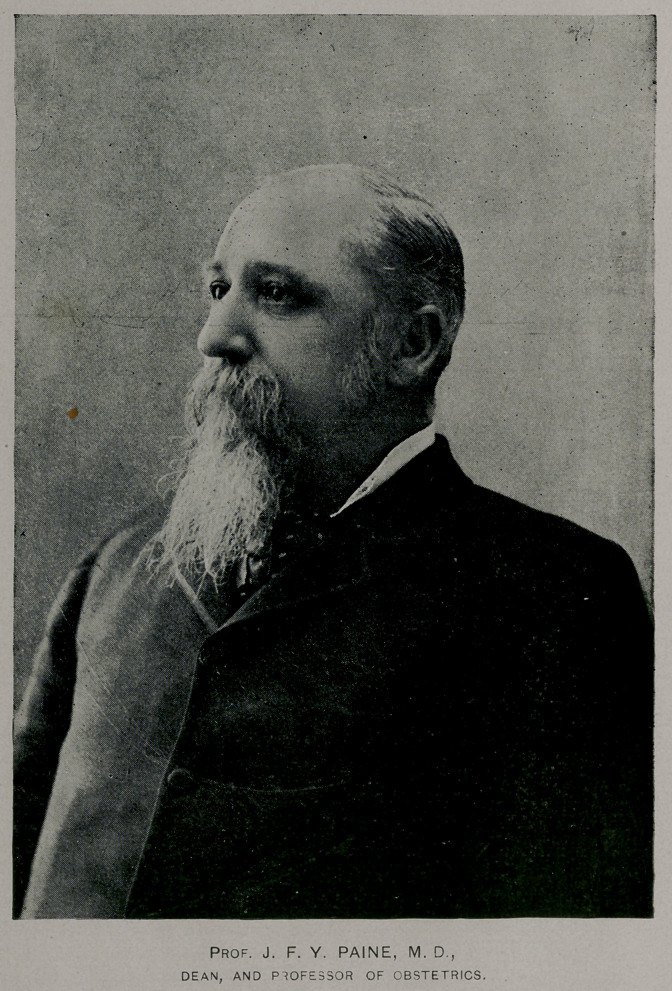


**Figure f2:**
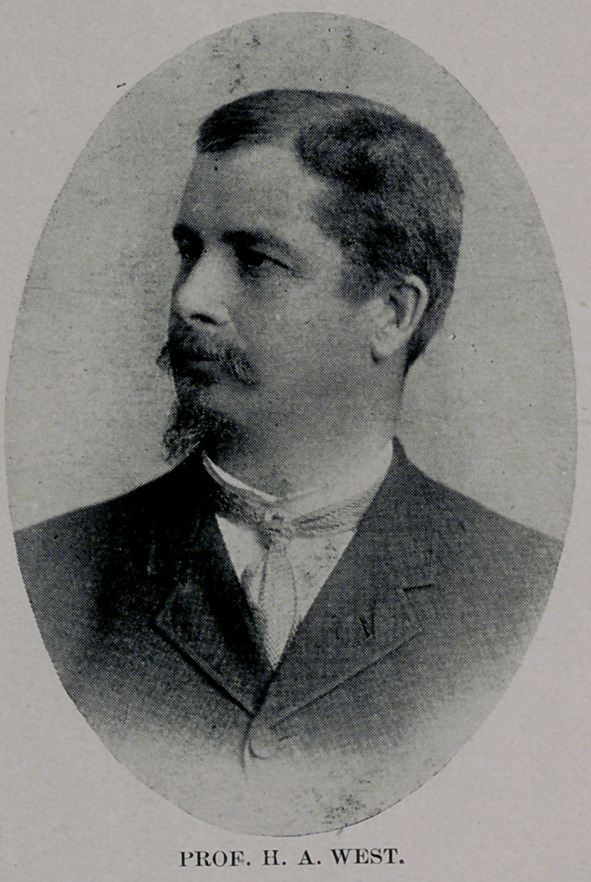


**Figure f3:**
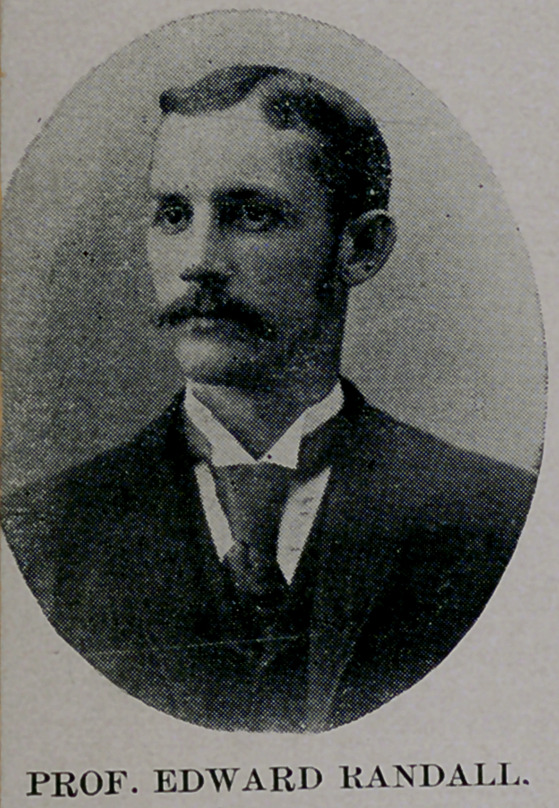


**Figure f4:**
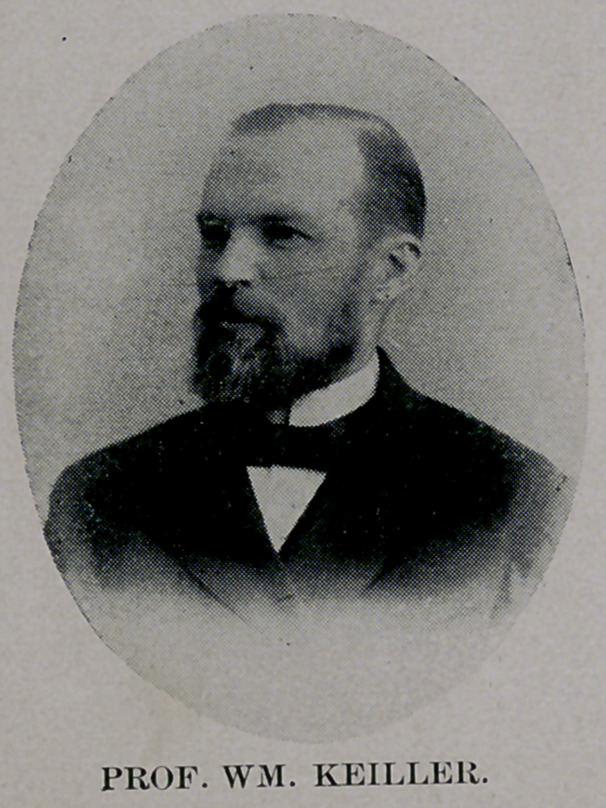


**Figure f5:**
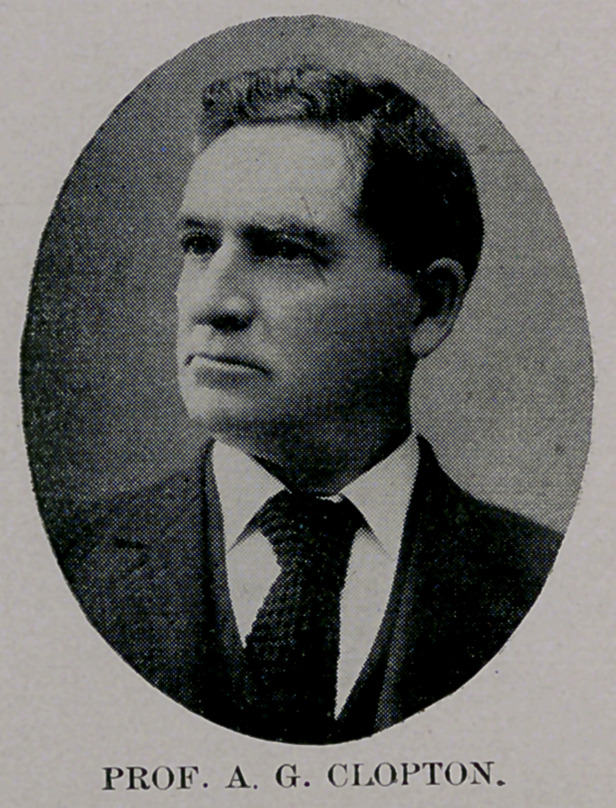


**Figure f6:**
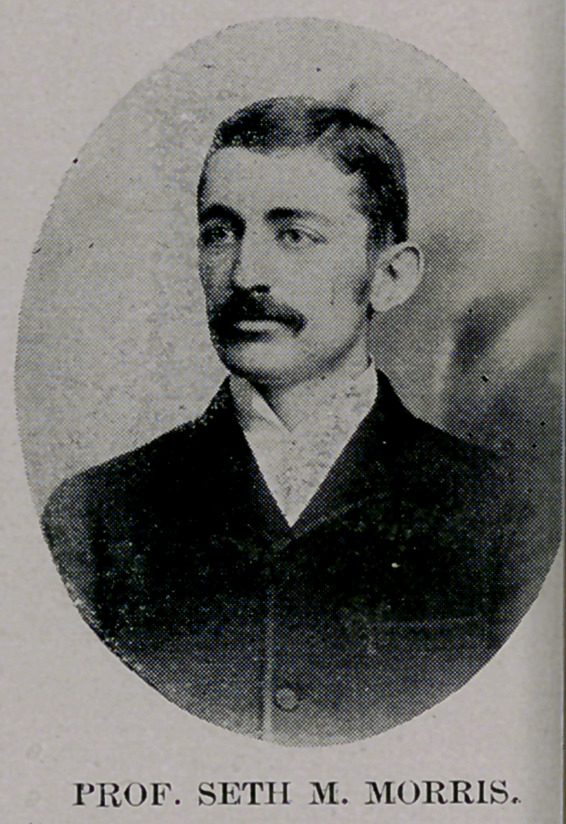


**Figure f7:**
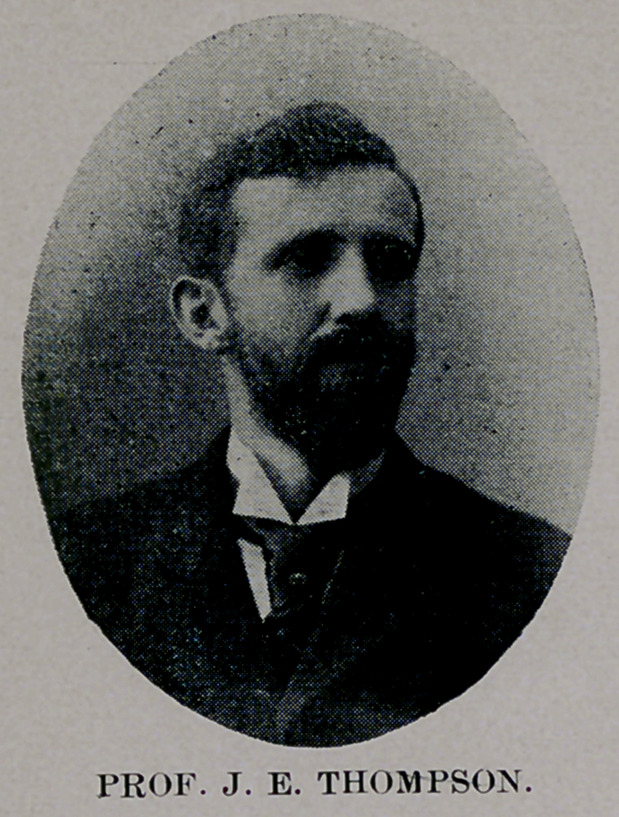


**Figure f8:**
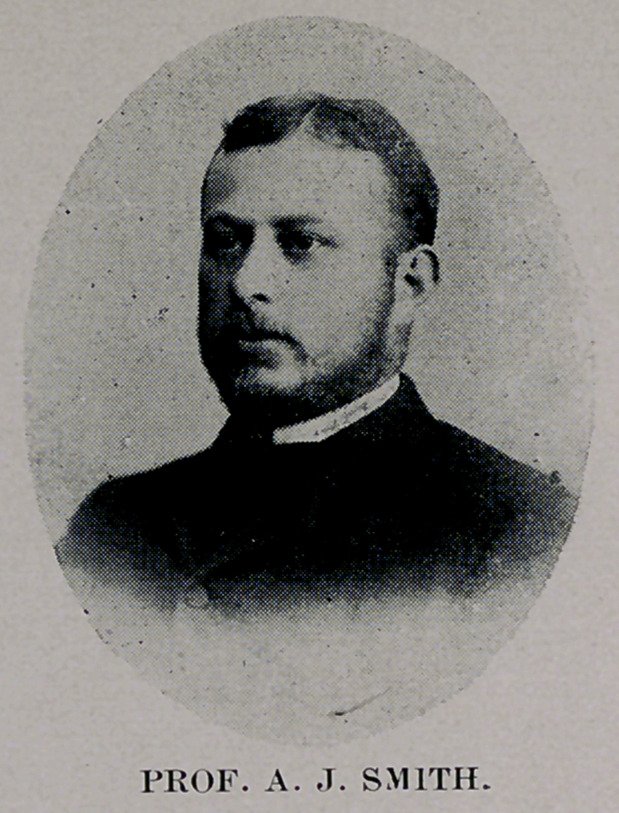


**Figure f9:**