# Errata

**Published:** 1892-08

**Authors:** 


**Errata.**—In our July number there occurred several provoking errors; the name of Dr. R. C. Hodges was spelled Hodge; Dr. H. P. Cooke's name was spelled "Cook." In Dr. H. Atchison West's name the middle name was spelled "Atcheson" and his place of nativity, Russell's Cave, was printed "Rupell's Cave." The word spectator was printed "speculator," and "extended trip" was printed "intended trip," etc. "Excuse these tears."

